# Shifting the gaze on implementation: examining the association between the implementation of tobacco control laws and prevalence of tobacco using data from a nationally representative survey

**DOI:** 10.1186/s12889-023-16780-8

**Published:** 2023-10-11

**Authors:** Pragati B. Hebbar, Upendra Bhojani, Onno van Schayck, Giridhara Babu, Vivek Dsouza, Gera E. Nagelhout

**Affiliations:** 1 Institute of Public Health Bengaluru, Bangalore, Karnataka India; 2 Department of Health Promotion, Maastricht University (CAPHRI), Maastricht, The Netherlands; 3 Department of Family Medicine, Maastricht University (CAPHRI), Maastricht, The Netherlands; 4 Epidemiology, IIPH-H, Bangalore Campus, Public Health Foundation of India, IIPH-H, Bangalore, Karnataka India; 5 IVO Research Institute, The Hague, The Netherlands

**Keywords:** Tobacco use, Tobacco control policies, Implementation, Implementation research, COTPA, NTCP, India

## Abstract

**Background:**

Tobacco use and the associated health burden is a cause of concern in India and globally. Despite several tobacco control policies in place, their sub-optimal and variable implementation across Indian states has remained a concern. Studies evaluating the real-world implementation of policies such as Cigarettes and Other Tobacco Products (COTPA) or National Tobacco Control Program (NTCP) in India and its association with reductions in tobacco use are limited. In this paper, we analyse data from a nationally representative survey to examine how policy implementation is associated with the tobacco use prevalence in India.

**Methods:**

We analysed data from the Global Adult Tobacco Survey (GATS 2016–17) India using multivariable logistic regression. The dependent variables were the use of smoked tobacco, smokeless tobacco, and tobacco in any form. The independent variables were proxies of implementation of the COTPA and the NTCP. We followed a step-wise backward elimination technique to reach the best fit models.

**Results:**

People exposed to no-smoking signages had lower odds of using tobacco (OR = 0.70, *p* < 0.001). People exposed to second-hand smoke (OR = 1.51, *p* < 0.001) and tobacco product advertisements (OR = 1.23, *p* < 0.001) had greater odds of using tobacco. Exposure to tobacco advertisements was associated with higher odds of using smokeless tobacco (OR = 1.23, *p* < 0.001), and smoked (OR = 1.33, *p* < 0.001) forms of tobacco.

**Conclusion:**

We find significant association between the implementation of tobacco control laws/programs and tobacco use in India. Our findings highlight the potential that policy implementation holds in reducing population-level tobacco use thus drawing attention towards the implementation phase of policies. The findings have implications on prioritising enforcement of specific tobacco control measures such as smokefree laws, modifying COTPA signages to encompass all tobacco products including against smokeless tobacco use and strengthening indirect advertising restrictions. Future research could focus on developing and validating predictors specific to policy implementation to support policy evaluation efforts.

**Supplementary Information:**

The online version contains supplementary material available at 10.1186/s12889-023-16780-8.

## Contributions to the literature

- Those who were aware about tobacco-related health harms and exposed to prohibitory signages were less likely to use tobacco products whereas exposure to second-hand smoke increased the likelihood of tobacco use suggesting the need to strengthen smoke-free provisions.
- It suggests tailored interventions such as signages for restricting smokeless tobacco use given the varied nature of tobacco use and India’s geographical complexity
- It provides direction for future studies, to focus on developing predictors specific to policy implementation for the common provisions such as smoke-free public places, youth access restriction, pictorial health warnings and advertisement bans and on scaling up implementation across varied contexts.

## Introduction

The prevalence of tobacco use among Indians is high, with 28.6% of adults using tobacco, which translates to about 266.8 million individuals using tobacco in one form or the other. Every third adult in rural areas and every fifth adult in urban areas of India currently uses tobacco. About 10.7% adults (99.5 million) currently smoke tobacco while 21.4% adults (199.4 million) currently use smokeless tobacco [1]. The burden of tobacco-related death and disease is concerning, with over 1.3 million deaths per year. In addition, the economic costs of tobacco use far outweigh the government spending on health [2–4].

India was one of the early signatories of the WHO Framework Convention on Tobacco Control (FCTC) and has enacted several tobacco control policies at the state and national levels [5]. The Cigarettes and Other Tobacco Products (Prohibition of advertisement and regulation of trade and commerce, production, supply and distribution) Act (COTPA), 2003 is the comprehensive tobacco control law applicable to all Indian states and Union territories in India. The major enforceable provisions of COTPA are the prohibition of smoking in public places, prohibition of direct and indirect advertisement of tobacco products, restricting sales to and by minors and within 100 yards of any educational institutes and mandating specified pictorial health warnings on tobacco product packages [6]. In 2008, the Government of India launched the National tobacco control program (NTCP) to support tobacco control efforts with a specific focus on COTPA implementation.

The implementation of the WHO-FCTC and its association with reduced tobacco consumption have been studied in India and several other countries [7, 8]. Models projecting the effects of tobacco control policies have found reductions in tobacco use prevalence on global and country levels. Despite evidence on the effectiveness of certain policy measures such as increased taxation only six countries of ﻿Argentina, Chile, Cuba, Egypt, Palau and San Marino have policies in line with WHOs recommended 70% excise tax share. Countries like Brazil have shown a reduction in smoking prevalence between 2009 to 2017 through sustained tobacco control efforts [9, 10] The impact of tobacco control policies on the prevalence of smoking and quit ratios in the European Union countries have been studied using a tobacco control scale (TCS) developed to quantify the implementation of tobacco control policies. The study shows the association of tobacco control policy implementation with lower smoking prevalence and higher quit ratios [11]. A recent study found that countries with a better preparedness for policy measures and a high burden of tobacco use showed significant reductions in smoking prevalence [12].

The available evidence is mostly in the form of predictive models, which have contributed to raising attention on the aspect of implementation. However, studies evaluating the real-world implementation of policies such as COTPA or NTCP in India and its association with reductions in tobacco use are limited. Studies have largely documented the awareness and compliance related to COTPA among certain subgroups of individuals and its specific provisions [13–16]. While the prevalence of tobacco has reduced over the decades in India; it has varied across states and sub-populations. Similarly, the implementation of policies has also varied across Indian states [17–19]. Over the past two decades several important policy developments have occurred in India such as the increase of the size of pictorial health warnings on tobacco products, ban of electronic cigarettes, introduction and scaling of quit lines to support cessation.

In this study, we aim to understand whether the implementation of tobacco control laws is associated with tobacco consumption at the population level in India. We hypothesize that better implementation of tobacco control policies will reduce the prevalence of tobacco use. There are several tobacco products in India, broadly classified as smoked and smokeless. Hence, we aimed to examine the association of implementation with tobacco consumption in any form, smoked and smokeless forms.

## Methods

We used cross-sectional data from round 2 (2016 -17) of the Global Adult Tobacco Survey (GATS) India [1]. As we used anonymized data from datasets available in public domain an ethics clearance was not applicable. These data sources were part of the phase 1 of the larger study protocol submitted to the Institutional Ethics Committee at the Institute of Public Health Bengaluru and had received an exemption. The supplementary file 1 explains the STROBE checklist items. GATS is a nationally representative face-to-face and interviewer lead survey among individuals aged 15 years and older. The GATS used a multi-stage, geographically clustered sample design and was carried out in 30 states and two union territories of India producing data representative of each state and union territory. The GATS-2 survey contains data from a total of 74,037 completed interviews. As the sample size of individuals between the age group of 15 – 18 years was relatively small of 3112 individuals, we excluded this subgroup. The final dataset for this study included 70,925 observations for people aged 18 or older.

### Measures

The outcome variables, predictor variables and the background characteristics were chosen for the study based on theoretical knowledge of earlier studies and the availability of proxy variables of implementation of the Cigarettes and Other Tobacco Products Act (COTPA) 2003 and the National Tobacco Control Programme (NTCP) within the GATS-2 dataset [20].

Owing to the varied nature of tobacco products and their usage patterns in India, we used three measures for the outcome variable of the prevalence of tobacco use for this study. The first was ‘tobacco use’, which included responses of daily or less than daily (1) versus not at all (0) for any tobacco product. Dual tobacco users were not excluded. The second was ‘smoked tobacco use’, which included responses of daily or less than daily (1) versus not at all (0). The third was ‘smokeless tobacco use', which included daily or less than daily (1) versus not at all (0) responses.

We chose predictor variables that assessed some aspects of implementing the three key provisions of COTPA and the awareness-raising function of NTCP. The predictor variable for Sect. 4 of COTPA [prohibition of smoking in public places] included exposure to secondhand smoke (yes or no) at any public place and noticing ‘no-smoking’ signage (yes or no). The predictor variable for Sect. 5 of COTPA [prohibition of all forms of direct and indirect tobacco product advertisements except at the point of sale] was exposure to tobacco product advertisements in the past 30 days (yes or no). Analysis of Sect. 6 [restriction of access to tobacco products by youth] related indicators were not included in this study as we only included observations for people aged 18 or above. The predictor variable for Sects. 7 and 8 of COTPA [mandated pictorial health warnings on tobacco products] included exposure to pictorial health warnings on tobacco product packages (yes or no). For the NTCP, we used three predictor variables assessing awareness about the harms of secondhand smoke exposure, harms of smoking, and harms of using smokeless tobacco communicated to the public through various media (yes or no) as the implementers of these are largely similar to those responsible for implementing COTPA sections, unlike the administrators of centralized quit lines. Also, the mcessation and quit line introduced by the government are relatively recent in 2016 compared to the COTPA 2003 and NTCP 2007–08. Hence, we did not include the cessation component of NTCP in this analysis.

We included background characteristics of age, sex, education, occupation, religion, and caste, as earlier studies have shown their association with tobacco use. Age was used as a continuous variable [21–23]. Sex had two responses of male and female. Education included four responses: (i) no formal schooling, (ii) up to primary school completed, (iii) up to secondary school completed, and (iv) college/university completed or postgraduate degree completed. Occupation included four categories: (i) employed (includes government, non-government, and self-employed), (ii) daily wage / casual labourer, (iii) homemaker, and (iv) unemployed (includes retired, individuals who are able and unable to work but still unemployed and students). Religion was categorized into four groups representing Hindus, Muslims, Christians and others. Caste was categorized into four groups: scheduled caste, scheduled tribe, other backward caste, and unreserved. Details of these categorization can be found in the GATS 2 full report and the codebook [1].

### Data analysis

We downloaded the data set of GATS round 2 of India from the Global Tobacco Surveillance System Data website of the Centers for Disease Control and Prevention. We used STATA version 15.1 SE (StataCorp, Texas, USA) for this study. We carried out bi-variate associations between outcome and predictor variables. We included those predictor variables that had a significant association with the outcome variable at a *p*-value of < 0.05 and included those in the first logistic regression model.

After running the first model, ensuring the goodness of fit and ruling out collinearity, we followed step-wise backward elimination of variables from the first model. We compared the newer models with the earlier ones regarding their goodness of fit. We arrived at models that showed no further improvement from previous models. We present the three final models with the adjusted odds ratio, 95% confidence interval and p-values.

## Results

### Sample characteristics

The GATS – 2 comprised 70,925 observations of respondents above 18 years of age. The demographic characteristics of the sample are presented in Table 1. The age of the respondents ranged from 18 to 110, the mean age was 40.46 years. The prevalence of tobacco use in any form was 30.48%, the prevalence of current tobacco smoking was 11.42%, and current smokeless tobacco use was 22.73%. In the predictor variables, 58.22% of adults noticed the no smoking signage and 36.2% of adults were exposed to secondhand smoke (SHS) at any public place, 18.92% of adults noticed tobacco advertisements in the last 30 days, whereas 57.11% of adults noticed pictorial health warnings on tobacco product packages.

**Table 1:** Demographic characteristic of the study dataset

	**N**	**%**
**Sex**		
Female	38,629	54.46
Male	32,296	45.54
**Education**		
No formal schooling	18,361	25.91
Up to primary school completed	15,968	22.53
Up to secondary school completed	20,231	28.55
Pre University and up to University degree completed	16,306	23.01
**Occupation**		
Employed (includes govt., non-govt. and self-employed)	23,425	33.04
Daily wage/ casual labourer	13,566	19.14
Homemaker	25,530	36.01
Unemployed (includes retired, individuals who are able and unable to work but still unemployed and students)	8374	11.01
**Religion**		
Hindu	51,821	73.1
Muslim	8296	11.7
Christian	6838	9.65
Others	3931	5.55
**Caste**		
Scheduled caste	12,272	17.41
Scheduled tribe	11,635	16.5
Other backward caste	26,146	37.08
None of these	20,451	29.01

### Tobacco use in any form

Table 2 depicts the logistic regression model using tobacco use in any form as the outcome indicator. Those aware of dangers of smoking (OR = 0.85, *p* = 0.048), smokeless tobacco use (OR = 0.75, *p* < 0.001) and those who reported to be exposed to no-smoking signages (OR = 0.70, *p* < 0.001) were less likely to use tobacco. Those exposed to SHS (OR = 1.51, *p* < 0.001) and to tobacco advertisements (OR = 1.23, *p *< 0.001) were more likely to use tobacco. These findings marked positive associations between implementation of the COTPA and less tobacco use. Defying this trend, we found that those who reported being aware of the dangers of SHS had higher odds of using tobacco (OR = 1.08, *p* < 0.001).

**Table 2:** Logistic regression model using predictors of current tobacco use in any form

Current tobacco use	Odds ratio	*P*-value	95% Conf. interval	
Awareness on dangers of smoking	0.85	**0.048**	0.72	1.00
Awareness on dangers of smokeless tobacco use	0.75	** < 0.001**	0.67	0.85
Awareness on dangers of exposure to SHS	1.08	**0.001**	1.03	1.13
No smoking signage noticed	0.70	** < 0.001**	0.64	0.76
Exposed to SHS	1.51	** < 0.001**	1.40	1.64
Tobacco product advertisement noticed	1.23	** < 0.001**	1.11	1.35
**Age**	1.01	** < 0.001**	1.01	1.01
**Sex**				
Female	-	**-**	-	-
Male	4.01	** < 0.001**	3.48	4.62
**Education**				
No formal schooling	-	-	-	-
Up to primary school	0.94	0.474	0.81	1.11
Up to secondary school	0.55	** < 0.001**	0.47	0.64
Higher secondary to post graduation	0.25	** < 0.001**	0.21	0.29
**Occupation**				
Employed (govt./ non-govt./self)	-	**-**	-	-
Daily wager	1.23	** < 0.001**	1.11	1.37
Homemaker	0.60	** < 0.001**	0.50	0.73
Unemployed (including retired and students	0.47	** < 0.001**	0.41	0.54
**Religion**				
Hindu	-	**-**	-	-
Muslim	1.21	**0.004**	1.06	1.37
Christian	1.24	**0.007**	1.06	1.44
Others	0.59	** < 0.001**	0.48	0.73
**Caste**				
Unreserved category	-	-	-	-
Scheduled caste	1.17	**0.031**	1.01	1.34
Scheduled tribe	2.80	** < 0.001**	2.40	3.28
Other backward caste	0.84	**0.001**	0.76	0.93

- Is used to indicate reference categories for the analysis

### Smoked tobacco use

Table 3 depicts the analysis for smoked tobacco use. Those who were exposed to no-smoking signages (OR = 0.86, *p* = 0.003) and were aware of the dangers of smokeless tobacco (OR = 0.73, *p* < 0.001) had lower odds of smoking tobacco. Those exposed to SHS (OR = 1.66, *p* < 0.001) or to tobacco advertisements (OR = 1.33, *p* < 0.001) had greater odds of smoking tobacco.

**Table 3:** Logistic regression model using predictors of current smoked tobacco use

Current smoked tobacco use	Odds ratio	P-value	95% Conf. interval	
Awareness on dangers of smokeless tobacco use	0.73	** < 0.001**	0.65	0.82
No smoking signage noticed	0.86	**0.003**	0.78	0.95
Exposed to SHS	1.66	** < 0.001**	1.51	1.83
Tobacco product advertisement noticed	1.33	** < 0.001**	1.19	1.49
**Age**	1.01	** < 0.001**	1.01	1.01
**Sex**				
Female	-	**-**	-	-
Male	9.85	** < 0.001**	7.80	12.44
**Education**				
No formal schooling	-	**-**	-	-
Up to primary school	0.90	0.224	0.77	1.06
Up to secondary school	0.55	** < 0.001**	0.46	0.64
Higher secondary to post graduation	0.29	** < 0.001**	0.24	0.35
**Occupation**				
Employed (govt./ non-govt./self)	-	**-**	-	-
Daily wager	1.19	**0.004**	1.06	1.33
Homemaker	0.86	**0.310**	0.64	1.15
Unemployed (including retired and students	0.58	** < 0.001**	0.49	0.69
**Religion**				
Hindu	-	**-**	-	-
Muslim	1.18	**0.026**	1.02	1.37
Christian	1.98	** < 0.001**	1.67	2.34
Others	0.91	0.421	0.71	1.15
**Caste**				
Unreserved category	-	-	-	-
Scheduled caste	1.02	0.816	0.86	1.21
Scheduled tribe	1.76	** < 0.001**	1.48	2.09
Other backward caste	0.80	** < 0.001**	0.70	0.90

- Is used to indicate reference categories for the analysis

### Smokeless tobacco use

Table 4 depicts associations with smokeless tobacco use. Those aware of dangers of using smoking tobacco products (OR = 0.71, *p* < 0.001) and those who noticed no-smoking signages (OR = 0.70, *p* < 0.001) were less likely to use smokeless tobacco products. Those exposed to SHS (OR = 1.32, *p* < 0.001) or to tobacco advertisements (OR = 1.23, *p* < 0.001) were more likely to use smokeless tobacco. However, those aware of the dangers of SHS exposure also showed greater odds (OR = 1.15, *p* < 0.001) of smokeless tobacco use.

**Table 4:** Logistic regression model using predictors of current smokeless tobacco use

Current tobacco use	Odds ratio	P-value	95% Conf. interval	
Awareness on dangers of exposure to SHS	1.15	** < 0.001**	1.10	1.20
No smoking signage noticed	0.70	** < 0.001**	0.64	0.76
Exposed to SHS	1.32	** < 0.001**	1.21	1.44
Tobacco product advertisement noticed	1.23	** < 0.001**	1.11	1.36
Awareness on dangers of smoking	0.71	** < 0.001**	0.62	0.82
**Sex**				
Female	-	**-**	-	-
Male	1.61	** < 0.001**	1.40	1.85
**Education**				
No formal schooling	-	-	-	-
Up to primary school	1.03	0.72	0.88	1.20
Up to secondary school	0.79	** < 0.001**	0.68	0.92
Higher secondary to post graduation	0.37	** < 0.001**	0.32	0.44
**Occupation**				
Employed (govt./ non-govt./self)	-	-	-	-
Daily wager	1.04	0.490	0.93	1.16
Homemaker	0.50	** < 0.001**	0.41	0.61
Unemployed (including retired and students	0.50	** < 0.001**	0.43	0.58
**Religion**				
Hindu	-	-	-	-
Muslim	1.11	0.150	0.97	1.27
Christian	0.90	0.200	0.77	1.06
Others	0.65	** < 0.001**	0.52	0.81
**Caste**				
Unreserved category	-	**-**	-	-
Scheduled caste	1.28	** < 0.001**	1.11	1.49
Scheduled tribe	2.25	** < 0.001**	1.91	2.64
Other backward caste	1.02	0.740	0.91	1.14

- Is used to indicate reference categories for the analysis

## Discussion

In this study, we explored the association between the implementation of tobacco control laws/programs and the prevalence of tobacco use in India using data from a nationally representative survey. We found that several proxy indicators of implementation are associated with the prevalence of tobacco consumption.

The models support the hypothesis that better implementation of COTPA and NTCP will reduce tobacco use prevalence. The implementation of provisions like the prohibition of smoking in public places (thereby reducing exposure to SHS) is shown to be significantly associated with lower tobacco use prevalence. Similarly, exposure to no-smoking signages in public/workplaces is associated with lesser odds of using smoked and smokeless tobacco products. Studies from India and other LMICs have shown the need for improving the enforcement of smoke-free provisions to protect non-smokers from exposure to SHS [24–27]. A recent study in Malaysia revealed that males and younger adults were exposed to SHS in smoking non-restricted areas. Similarly, in Bangladesh, exposure to SHS was high in settings having partial bans [24, 25]. Our study shows that those exposed to SHS are more likely to use tobacco products, and consequently supports the need to improve the implementation of smoke-free provisions. In India's context of high smokeless tobacco use, we argue that mandating no-spitting signages in public/workplaces along with the currently mandated no-smoking signages as per COTPA might prove useful in reducing smokeless tobacco use. This could build on the formal guidance by the Indian Council of Medical Research issued in the early part of the COVID-19 pandemic, communicating the importance of avoiding spitting in public places [28].

Exposure to tobacco product advertisements was associated with a higher chance of tobacco use in all three models, suggesting the need to focus on curbing advertisements for tobacco products. A study examining the impact of advertising on adult smokers’ awareness of tobacco products in Malaysia and Thailand revealed low awareness of tobacco products and marketing in Thailand, where restrictions were well implemented, but significantly higher in Malaysia [29]. Indirect advertisements through brand stretching are currently rampant in India [30, 31] This study supports the call to restrict the indirect advertising and marketing of tobacco products as a potential aid in reducing the prevalence of tobacco consumption.

The exposure to pictorial health warnings on tobacco products did not feature in the final models, indicating a lack of association with tobacco use prevalence. This appears somewhat in contrast with an observation from the same GATS-2 survey. Those tobacco users who noticed the pictorial health warnings, 61.9% of cigarette smokers, 53.8% of bidi smokers and 46.2% of smokeless tobacco users, reported thinking about quitting tobacco use because of warning labels [1, 32]. Possible explanations for this finding are that (1) the exposure to pictorial health warnings on tobacco products is likely concentrated among tobacco users as the non-users are less likely to be exposed to these labels, and (2) ‘thinking about quitting’ is a desirable step but does not assure successful quitting or abstinence. Hence, our finding cannot be seen as a verdict on the effectiveness of the warning labels.

Awareness of the dangers of using smoked and smokeless tobacco products was related to a lower likelihood of using any form of tobacco and smoked tobacco. These findings suggest the relevance of increasing awareness of the harms of tobacco, supporting the need for awareness generation activities carried out under the NTCP. In models of any form of tobacco use and smokeless tobacco; the direction of change for awareness about the harms of exposure to SHS indicates those aware of the harms are more likely to use tobacco products. This could possibly indicate reversed causality where smokeless tobacco users being target of awareness campaigns have become more aware of dangers of tobacco including SHS compared to non-users. However, the effect size is very small (0.8). The GATS 2 report also shows that 67.3% of adults noticed anti-smokeless tobacco information, whereas 76% noticed anti-smoking information [1]. This further suggests the need to focus on raising awareness against smokeless tobacco use and revisiting and revising the messaging on the harms of SHS exposure, probably more tailored to local perceptions and improving the message delivery to reach relevant audiences [19].

There are several strengths and limitations of this study. A strength is that this study is one of the first studies to explore the association of tobacco control policy implementation with the prevalence of different types of tobacco use in India. The survey this study analyses is nationally representative, with standardized methodology and tobacco-specific, providing reliable and representative data for the country. A limitation is the bias of self-reported nature of data collected through the GATS survey. However, studies from India have shown that self-reporting is still a relatively reliable measure of actual tobacco use [33]. In this study, we analyze the association of COTPA 4, 5 and 7 and the awareness raising component of NCTP as the proxy indicators for these were available in the GATS. But beyond these tobacco control measures, there are other national, state and local level tobacco control measures in place which have not been included in this study, such as bans on smokeless tobacco products and bans on the sale of single cigarette sticks, bans on e-cigarettes etc. The proxy indicators of implementation selected from the GATS dataset need to be further validated to ensure they provide a good estimate of the level of implementation. For example, awareness of pictorial warning labels may not measure implementation of this policy but simply exposure to packs and remembering the warning labels. Therefore, updating and critically validating the Tobacco Control Scale that addresses its existing limitations can be one of the future research direction [34]. Another important limitation is the inability to include the cessation component of NTCP into this study because of the reasons explained above. Lastly, our cross-sectional study design does not ascertain causal and unidirectional links between predictors and outcomes. Hence, at best, it provides rationale and guidance for further studies that can specifically examine causal links.

## Conclusion

COTPA and NTCP are comprehensive tobacco control policies and programs in India, and effective implementation may lead to a reduction in tobacco use prevalence across Indian states. Our study calls attention to the implementation phase of tobacco control policies beyond the development and adoption of policies. It highlights the potential that policy implementation holds in reducing population-level tobacco use in line with other studies. Our results have implications on prioritising enforcement of specific tobacco control measures such as smokefree laws, consider modifying COTPA signages to include all tobacco products including against smokeless tobacco use and strengthen indirect advertising restrictions. We need greater focus on researching what helps in scaling up implementation of tobacco control policies across contexts in India. Future research could focus on developing and validating predictors specific to policy implementation to support policy evaluation efforts including in the next round of the GAT survey. Also, studies should examine causal links between the implementation of policies and tobacco prevalence outcomes.

### Supplementary Information

**Additional file 1.**

## Data Availability

The datasets generated and/or analysed during the current study are available in the Global Tobacco Surveillance System Data (GTSSData) repository, https://nccd.cdc.gov/GTSSDataSurveyResources/Ancillary/DataReports.aspx?CAID=2
